# *Theileria terrestris* nov. sp.: A Novel *Theileria* in Lowland Tapirs (*Tapirus terrestris*) from Two Different Biomes in Brazil

**DOI:** 10.3390/microorganisms10122319

**Published:** 2022-11-23

**Authors:** Anna Claudia Baumel Mongruel, Emília Patrícia Medici, Ariel da Costa Canena, Ana Cláudia Calchi, Lívia Perles, Bianca Cardenal Balla Rodrigues, João Fabio Soares, Rosangela Zacarias Machado, Marcos Rogério André

**Affiliations:** 1 Vector-Borne Bioagents Laboratory (VBBL), Departamento de Patologia, Reprodução e Saúde Única—Faculdade de Ciências Agrárias e Veterinárias, Universidade Estadual Paulista, UNESP, Jaboticabal 14884-900, SP, Brazil; 2 Iniciativa Nacional para a Conservação da Anta Brasileira (INCAB), Instituto de Pesquisas Ecológicas (IPÊ), Campo Grande 79046-150, MS, Brazil; 3 Escola Superior de Conservação Ambiental e Sustentabilidade (ESCAS/IPÊ), Nazaré Paulista 12960-000, SP, Brazil; 4 IUCN SSC Tapir Specialist Group (TSG), Campo Grande 79046-150, MS, Brazil; 5 Laboratório de Protozoologia e Rickettsioses Vetoriais (ProtoZooVet), Departamento de Patologia Clínica Veterinária, Faculdade de Veterinária, Universidade Federal do Rio Grande do Sul, Porto Alegre 90010-150, RS, Brazil

**Keywords:** Piroplasmida, tapirs, Pantanal, Cerrado, theileriosis, wildlife

## Abstract

The low-land tapir (*Tapirus terrestris*) is the largest wild terrestrial mammal found in Brazil. Although *T. terrestris* has been already reported as a host of hemoparasites, the occurrence and genetic identity of Piroplasmida agents in this species is still cloudy. Although it is reported that *Theileria equi*, an endemic equid-infective agent in Brazil, is occurring in lowland tapirs, these reports are probably misconceived diagnoses since they are solely based on small fragments of 18S rRNA that may not achieve accurate topologies on phylogenetic analyses. The present study aimed to detect and investigate the identity of *Theileria* spp. in tapirs from Pantanal and Cerrado biomes. Blood-DNA samples from tapirs were screened for a partial (~800 bp) 18S rRNA gene fragment from Piroplasmida and 64 (64/122; 52.46% CI: 43.66–61.11%) presented bands of expected size. Samples were submitted to different protocols for molecular characterization, including near-full length 18S rRNA gene (~1500 bp), and the *ema-1* gene from *T. equi*. Eight sequences were obtained for extended fragments (1182–1473 bp) from the 18S rRNA gene. Moreover, three sequences from partial *cox-1* and five from partial *hsp70* gene were obtained. None of the samples presented amplifications for the *ema-1* gene. Phylogenetic and distance analyses from the 18S rRNA sequences obtained demonstrated a clear separation from tapirs’ *Theileria* spp. and *T. equi*. Phylogenetic analyses of *cox-1* and *hsp70* sequences obtained herein also showed a unique clade formed by tapir’s *Theileria* spp. *Theileria terrestris* sp. nov. is positioned apart from all other *Theileria* species in 18S rRNA, *cox-1*, and *hps70* phylogenetic analyses. This novel proposed species represents a new Piroplasmida clade, yet to be characterized regarding biological features, vectors involved in the transmission cycles, additional vertebrate hosts, and pathogenicity.

## 1. Introduction

Possibly, a larger number of parasites from Piroplasmida order may occur when compared to the number of vertebrate hosts that are capable of harboring these potential pathogens [1].

The low-land tapir (*Tapirus terrestris*) is the largest wild terrestrial mammal found in Brazil. Although *T. terrestris* has already been reported as a host of hemoparasites, such as *Trypanosoma* sp. [2] and hemoplasmas [3], the occurrence and genetic identity of Piroplasmida agents in this species is still incomplete.

In 2017, the molecular detection of piroplasms was reported in a single tapir from Mato Grosso do Sul State, Midwest Brazil. In this case, partial 18S rRNA (414 bp) amplification followed by sequencing and BLASTn analyses were conducted for nucleotide identity assessment, and BLASTn analysis indicated 98% identity with *Theileria equi* [4]. A few years later, *T. equi* was described as occurring in 11 Brazilian low land tapirs from the Amazon biome also based on the amplification of small fragments (392–475 bp) of the 18S rRNA gene and followed by maximum likelihood and Bayesian phylogenetic inferences [5].

The tick *Rhipicephalus microplus* is often associated as a vector of *T. equi* among horses in South America. However, due to decisive biological features, such as the fact that this tick species is a one-host tick and the lack of transovarilally transmission of *T. equi*, the role of *R. microplus* in *T. equi* cycle is still obscure [6]. *Amblyomma sculptum* has been reported infesting *T. equi*-positive horses in Brazil [7], including horses maintained in the Pantanal wetland areas [8]. However, *A. sculptum* was not able to transmit *T. equi* when feeding on infected horses under laboratory conditions [9]. Indeed, *A. sculptum* has been reported as a frequently found tick species in *T. terrestris* from Cerrado and Pantanal areas. *Rhipicephalus microplus* has also been reported in tapirs from these areas, albeit less frequently [10].

The present study aimed to investigate Piroplasmida in tapirs’ blood samples from two Brazilian biomes, namely Cerrado and Pantanal, as well as to describe a new species of *Theileria* by assessing its phylogenetic positioning using large fragments of the 18S rRNA gene and additional molecular markers.

## 2. Materials and Methods

### 2.1. Sampling

Blood samples were collected from tapirs from Cerrado and Pantanal biomes (municipalities of Nova Andradina and Aquiadana, Mato Grosso do Sul State, Brazil). In total, 125 blood samples were collected from 94 living-tapirs: 61 (61/94; 64.89% CI: 54.83–73.78%) from Pantanal wetland areas, 33 (33/94; 35.11% CI: 26.22–45.17%) from Cerrado, and eight road-killed tapirs (8/125; 6.40% CI: 3.28–12.12%), also from Cerrado biome, that were sampled during necropsy procedures, summarizing 102 sampled individuals in both areas [3]. Fast-stained (Romanowsky-type stain) blood smears were made with collected blood samples from living tapirs for investigation of hemoparasites’ inclusions in blood cells by light microscopy (Olympus BX43, Olympus Corporation, Tokyo, Japan) and images were captured using a lens’ attached camera (Olympus DP73, Olympus Corporation, Tokyo, Japan) and computer software (CellSens, Olympus Corporation, Tokyo, Japan).

This study was approved by the Ethics Committee for Animal Experimentation of FCAV/UNESP (Faculty of Agricultural and Veterinary Sciences of the São Paulo State University) under protocol number 4558/20. The “Instituto Chico Mendes de Conservação da Biodiversidade (ICMBIO)” provided the required annual permits for the capture and immobilization of tapirs and collection of biological samples (SISBIO# 14,603). All protocols for the capture, anesthesia, handling, and sampling of tapirs have been reviewed and approved by the Veterinary Advisors of the Association of Zoos and Aquariums (AZA)—Tapir Taxon Advisory Group (TAG), and the Veterinary Committee of the IUCN SSC Tapir Specialist Group (TSG). Further information about study areas and samplings is described elsewhere [3].

### 2.2. DNA Extraction and PCR Protocols for Mammals’ Endogenous Genes

DNA extraction was performed using a commercial kit (InstaGene™ Matrix, Biorad^®^, Hercules, CA, USA) and following the manufacturers’ instructions. In order to ensure successful DNA extraction, a conventional PCR for the mammal-endogenous gene glyceraldehyde-3-phosphate dehydrogenase (*gapdh*) (450 bp fragment) [11] was performed in all samples. Negative samples in the abovementioned PCR protocol were subjected to a second protocol targeting a 227-bp fragment of the *irpb* interphotoreceptor retinoid-binding protein (*irpb)* gene [12]. Samples that did not yield amplicons in either of the used PCR protocols were excluded from the subsequent analysis.

### 2.3. Conventional and Nested PCR Protocols for Piroplasmida Detection and Molecular Characterization

Initially, DNA samples from tapirs’ blood previously positive in the endogenous genes-based PCR protocols were submitted to a screening nested-PCR protocol aiming to amplify a ~800 bp fragment from the 18S rRNA gene of Piroplasmida [13]. Positive samples were then submitted to PCR protocols targeting fragments of different genes (*cox-1* [14,15], *cox-3* [16,17], *hsp70* [18], *cytb* [16,17], *β-tubulin* [19], and the intergenic spacer-1/ITS-1 [20]) from Piroplasmida as well as a fragment (396 bp) of *ema*-1 gene from *Theileria equi* [21]. Nucleotide sequences of primers and references used for the amplification of these genes are described in Table 1. Samples that yielded strong bands on electrophoresis for the partial 18S rRNA gene were submitted to two additional protocols for amplification of full-length 18S rRNA (~1500 bp) [22,23,24]. All PCR were conducted in a final volume of 25 μL. Detailed reagent concentrations and thermal conditions of all protocols used in the present study can be found in Supplementary File S1.

Products obtained in PCR assays were separated by electrophoresis on a 1% agarose gel stained with ethidium bromide (Life Technologies™, Carlsbad, CA, USA) at 100 V/150 mA for 50 min, using 5 μL of amplified DNA per sample. The gels were imaged under ultraviolet light (ChemiDoc MP Imaging System, Bio Rad™, Hercules, CA, USA) using the Image Lab Software v4.1 (Biorad, Hercules, CA, USA). A map was constructed using QGis v. 3.26 software (http://qgis.org accessed on 26 October 2022) to illustrate the results obtained for partial 18S rRNA amplification from individual tapirs from each biome. 

### 2.4. Sequencing, Sequence Analysis and Phylogeny

Amplified products were purified using a commercial kit (Wizard^®^ SV Gel and PCR Clean-Up System, Promega, Madison, WI, USA) and sequenced using the BigDye™ Terminator v3.1 Cycle Sequencing Kit (Thermo Fisher Scientific™, Waltham, MA, USA) and ABI PRISM 310DNA Analyzer (Applied Biosystems™, Foster City, CA, USA) [25], at the “Centro de Recursos Biológicos e Biologia Genômica” (CREBIO, FCAV/UNESP, Jaboticabal, São Paulo, Brazil).

For sequencing of selected amplicons, the same primer pair from each PCR protocol or the primer pair from the 2° reaction of nested PCR assays were used, with exception for sequencing of extended 18S rRNA fragments. In the last case, primers used for sequencing [18,26] are described in Table 2. The obtained sequences were first submitted to a screening test using Geneious 11.1.3 software (http://www.geneious.com (accessed on 21 September 2021)) to evaluate the electropherogram quality and generate the consensus sequences. The BLASTn online program (National Center for Biotechnology Information, Bethesda, MD, USA) [27] was used to analyze the nucleotide sequences aiming to browse and compare with sequences from GenBank international database (http://www.ncbi.nlm.nih.gov/genbank (accessed on 21 September 2021)). Consensus sequences obtained in the current study and those retrieved from GenBank were aligned using the ClustalW software [28] via Bioedit v7.0.5.3 (http://www.mbio.ncsu.edu/BioEdit/bioedit.html, accessed on 3 July 2022) and also improved by an MAFFT alignment performed using GUIDANCE2 online server (http://ww.guidance.tau.ac.il, accessed on 3 August 2022) [29]. Phylogenetic inferences were based on Bayesian analysis via CIPRES online server (https://www.phylo.org/index.php/, accessed on 3 August 2022) [30]. The best-fit model was determined using jModeltest v2.1.6 via CIPRES online server (https://www.phylo.org/index.php/, accessed on 3 August 2022) [31].

Additionally, a distance-based analysis was performed using SplitsTree v4.14.6 (University of Tubingen, Tubingen, Germany) [32] to investigate the genetic relationship among extended 18S rRNA sequences detected in the present study and those previously deposited in GenBank.

## 3. Results

### 3.1. PCR for Endogenous Mammal’s Genes

Out of 125 DNA samples extracted from blood samples, 122 samples (122/125; 97.60% CI: 93.18–99.18%) from 99 individuals successfully amplified the target endogenous genes. Three samples did not present bands of expected size for both protocols and were excluded from further PCR analyses. Regarding the animals that DNA from blood samples was submitted to further analyses; 61 (61/99; 61.62% CI: 51.77–70.59%) were sampled in Pantanal biome, whereas 38 (38/99; 38.38% CI: 29.41–48.23%) were sampled in Cerrado biome.

### 3.2. Screening Nested PCR for Piroplasmida Partial 18S rRNA Gene (~800 bp)

Out of 122 analyzed samples, 64 (64/122; 52.46% CI: 43.66–61.11%) showed expected band sizes on electrophoresis based on the 18S rRNA gene nested PCR. The 64 positive samples were obtained from 56 animals (56/99; 56.57% CI: 46.74–65.90%). Considering the prevalence at each biome, 41 positive animals were sampled in Pantanal (41/61; 67.21% CI: 54.72–77.66%) and 15 from the Cerrado (15/38; 39.47% CI: 25.60–55.28%). Among positive samples from Cerrado biome, four (4/15; 26.67%; CI: 10.90–51.95%) were collected from road-killed animals. From Pantanal biome, six animals presented more than one positive sample during recaptures in different dates (in duplicates or triplicates), totalizing 48 (48/64; 75% CI: 63.18–83.99%) positive samples from this area. Regarding Cerrado biome, one animal presented more than one positive sample during recaptures in different dates (in duplicate), totalizing 16 (16/64; 25% CI: 16.01–36.82%) positive samples from this area.

**Figure 1:**
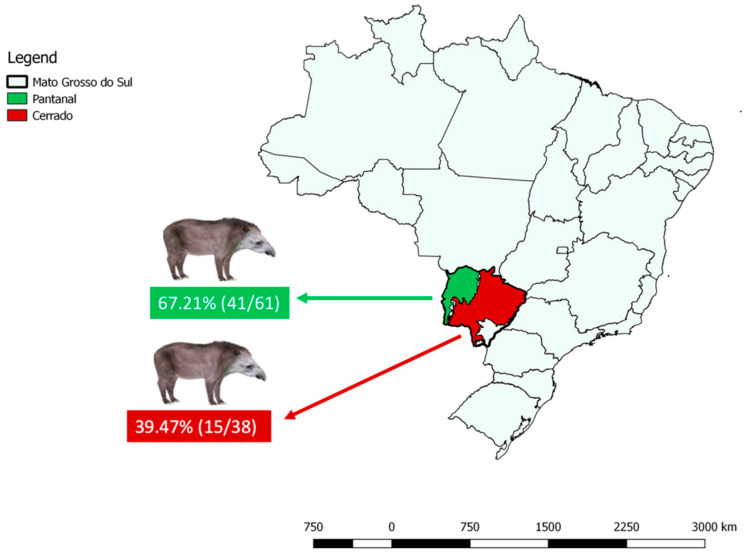
Number of tapirs from each biome that presented blood DNA samples with bands of expected sizes on agarose gel electrophoresis when tested for partial Piroplasmida 18S rRNA gene by nested PCR analysis [13] in Mato Grosso do Sul State. This map was constructed using QGis v. 3.26 software (http://qgis.org, accessed on 26 October 2022).

The results described above are summarized in Supplementary Files S2 and S3. The map (Figure 1) illustrates the number of tapirs that presented bands of expected sizes for the tested protocol.

### 3.3. Amplification of Molecular Markers for Additional Molecular Characterization of Piroplasmida

Twenty-one samples (21/64; 32.81% CI: 22.57–45.0%) presented fragments of different sizes for the *cox*-1 protocol and three sequences were successfully obtained. Although the protocol used a ~940 bp target fragment, the *cox-1* sequences obtained from tapir ranged from 354 to 410 bp (GenBank access numbers OP169682, OP169600, OP169601). For the amplification of *hsp70* gene, 28 (28/64; 43.75%; CI: 32.29–55.91%) samples showed amplification of expected size and five sequences were successfully obtained with sizes ranging from 687 bp to 782 bp (GenBank access numbers (GenBank access numbers OP376711, OP169596-OP169599). Regarding the amplification of the Intragenic Spacer 1 (ITS-1), while 38 samples (38/64; 59.38% CI: 47.15–70.54%) presented amplification for the tested protocol, amplicons showed different size bands. Although different samples were chosen for purification and sequencing attempts, none of them were successfully purified and sequenced.

No sample presented amplification in the PCR protocols targeting the *cox*-3, *cytb*, *β-tubulin* genes, and *T. equi ema*-1 gene. BLASTn analyses results from the obtained sequences are described in Supplementary File S4.

### 3.4. Amplification of Extended Sequences from the Piroplasmida 18S rRNA Gene (~1500 bp)

Fifteen samples that presented strong amplification bands on electrophoresis for partial 18S rRNA (~800 bp) were sequenced to confirm identity and submitted to other two PCR protocols targeting an extended sequence of 18S rRNA gene (~1500 bp). Out of 15 samples tested, eight presented bands of the expected size for the first protocol [23,24]. Samples that did not yield bands of the expected size for the first protocol were then submitted to the second protocol [22] and additional four positive samples were obtained. All positive samples for both protocols (*n* = 12) were sequenced and eight sequences were successfully obtained, presenting sizes ranging from 1182 to 1473 bp (GenBank access numbers (OP023828-OP023835). BLASTn analyses results demonstrated that sequences obtained presented nucleotide identities ranging from 95.23% to 95.53% with *Theileria* spp. sequences from GenBank (Supplementary File S4).

### 3.5. Phylogenetic Analyses

For all three molecular markers described below, clades were identified according to the phylogenetic study of Piroplasmida performed by Jalovecka et al. (2019) [33].

**Figure 2:**
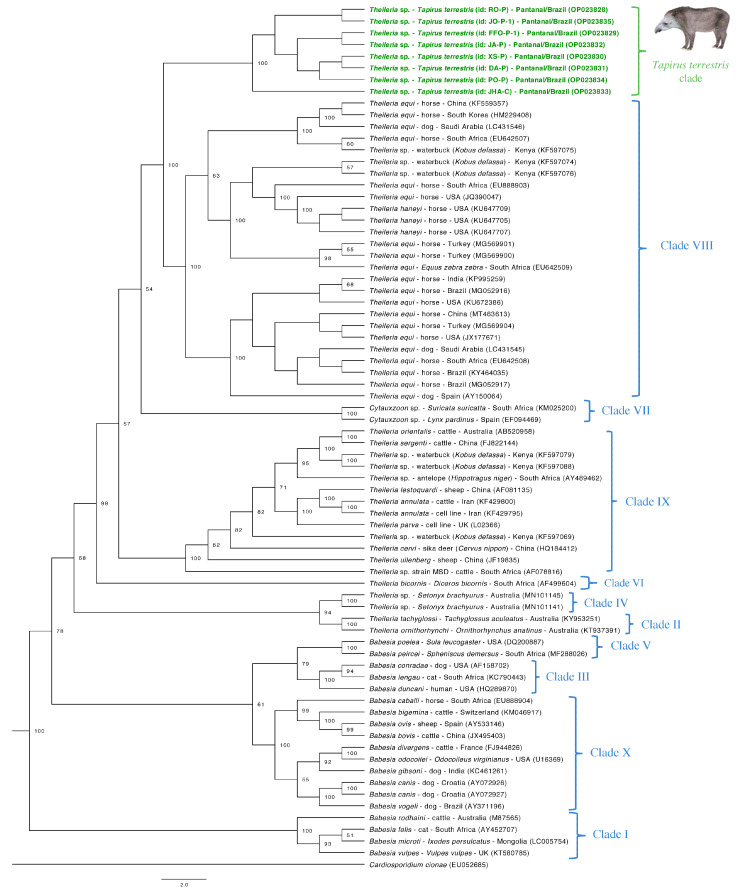
Phylogenetic tree based on the 18S rRNA gene from Piroplasmida. Tree was constructed by Bayesian inference using an alignment size of 1460 bp. Clades (I–X) were identified according to the phylogenetic study of Piroplasmida conducted by Jalovecka et al. (2019). Sequences from the present study are highlighted in green. Post probability values >50 appear in tree. Identifications of the phylogenetic groups as proposed by Jalovecka et al. (2019) are indicated next to taxa or groups.

The extended 18S rRNA sequences obtained herein were subjected to Bayesian inference phylogeny analysis (Figure 2) with more 65 homologue sequences from GenBank database. *Cardiosporidium cionae* (GenBank access n^o^. EU052685) was used as an outgroup. A total size of 1460 bp alignment was obtained and TrN+I+G was determined as the best-fit evolutionary model by Bayesian information criteria (BIC), using 10^7^ generations of MCMC (Monte Carlo Markov Chain), two independent runs, and 10% of burn-in. Piroplasmida 18S rRNA sequences obtained from tapirs’ blood samples in the present study formed a clade inside *Theileria* group, albeit separately from other *Theileria* species with high post-probability values (100).

**Figure 3:**
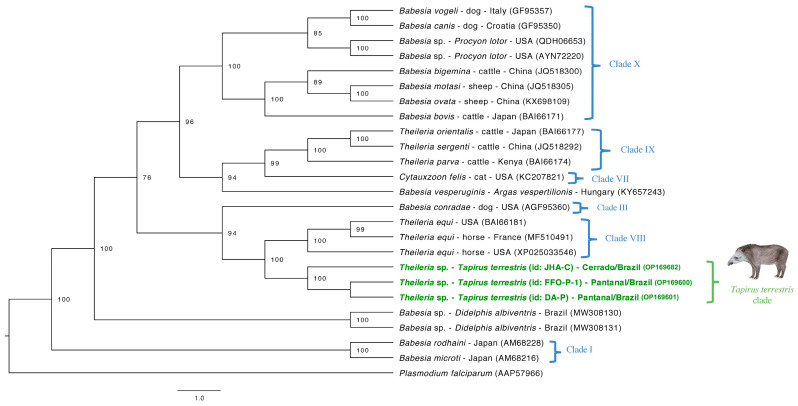
Phylogenetic tree based on partial *cox*-1 gene. Tree was constructed by Bayesian inference using an alignment size of 259 amino-acids. A sequence of *Plasmodium falciparum* (AAP57966) was used as outgroup. Sequences from the present study are highlighted in red. Post probability values >50 appear in tree. Clades (I, III, VII, VIII, IX, X) were identified according to the phylogenetic study of Piroplasmida conducted by Jalovecka et al. (2019) are indicated next to taxa or groups.

Regarding phylogenetic analysis based on the *cox-1* gene (Figure 3), a Bayesian inference phylogeny was performed with three sequences obtained in the present study and 21 homologous sequences retrieved from GenBank database. *Plasmodium falciparum* (AAP57966) was used as an external group. The nucleotide sequences were then transformed into amino acids using the ORFINDER software (https://www.ncbi.nlm.nih.gov/orffinder/ (accessed on 10 February 2022)) and a total alignment of 259 amino acids was set up. The analysis was performed using the GTR+G evolutionary model, 10^7^ generations of MCMC with two independent runs, and 10% burn-in. The sequences obtained for this fragment also grouped in a clade separated from other Piroplasmida species clades. The posteriori-probability value between lowland tapirs-associated *Theileria* and *Equus* group was 100.

**Figure 4:**
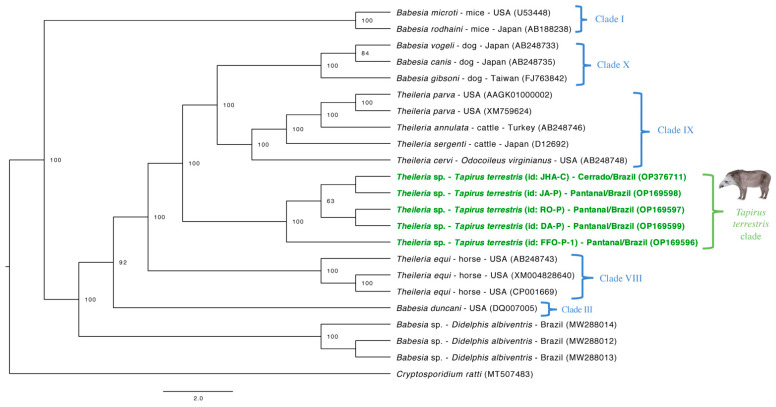
Phylogenetic tree based on partial *hsp70* gene. Tree was constructed by Bayesian inference using an alignment size of 690 bp. A sequence of *Cryptosporidium ratti* (MT507483) was used as outgroup. Sequences from the present study are highlighted in bold. Post probability values >50 appear in tree. Clades (I, III, VIII, IX, X) were identified according to the phylogenetic study of Piroplasmida conducted by Jalovecka et al. (2019) are indicated next to taxa or groups.

Phylogenetic analysis based on the five *hsp70* gene fragments obtained herein (Figure 4) was performed by Bayesian inference, with a total alignment of 690 bp, using 17 homologous sequences of the same gene and a sequence of *Cryptosporidium ratti* (MT507483) as an outgroup. The evolutionary model used for this analysis was F81+G, with 10^7^ generations of MCMC, two independent runs, and 10% burn-in. Sequences from the present study grouped in a separate clade from other Piroplasmida species. This time, the *Theileria* obtained from lowland tapirs presented as closer related with *Theileria* sensu stricto and *Babesia* sensu stricto groups than with *Equus* group, with a high posteriori-probability value (100).

### 3.6. Distance Analysis by Splistree Software

**Figure 5:**
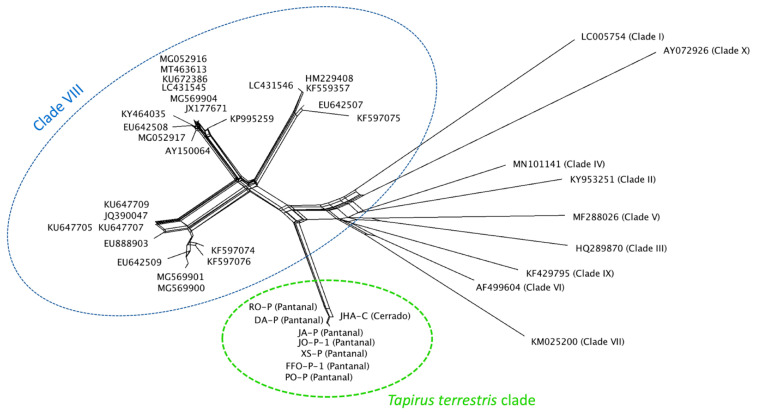
Splitstree network based on ‘Neighbor-net’ and ‘Uncorrected-p’ parameters, using an alignment of 1460 bp of the 18S rRNA. Identifications of the phylogenetic groups as proposed by Jalovecka et al. (2019) are indicated next to taxons or groups. Sequences from the present study are circled in green.

A distance analysis was performed with a total alignment of 1460 bp of the Piroplasmida 18S rRNA gene using Splistree with ‘Neighbor-net’ and ‘Uncorrected p-distance’ parameters (Figure 5). Taxons were identified according to the classification proposed by Jalovecka et al. (2019) [33]. Samples from the present study were circled in green. The extended sequences of 18S rRNA obtained herein formed a clearly separated group from *T. equi* group and other Piroplasmida species.

### 3.7. Blood Smears Analysis

**Figure 6:**
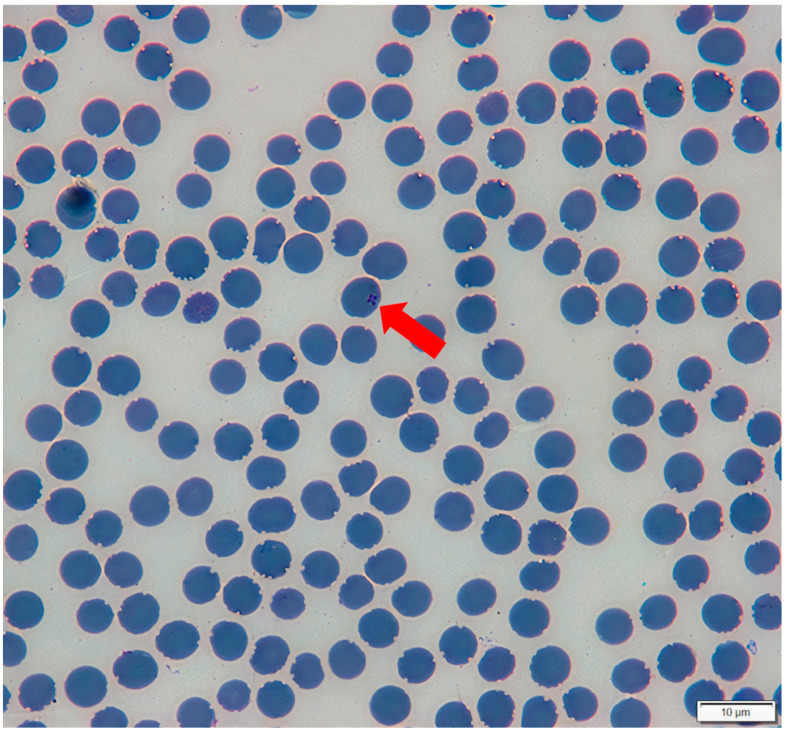
Maltese-Cross inclusions suggestive of *Theileria* spp. found in erythrocytes of *Tapirus terrestris* (indicated by the red arrow). Visualization was made by using a light microscope (1000×), during blood smear examination. The animal (ID: PO-P) was positive in a PCR protocol that amplify a fragment of approximately 1500 bp of the 18S rRNA gene from piroplasmids [23,24] in the present study.

Two blood smear samples from PCR-positive tapirs (ID: PO-P and FFO-P-1) presented inclusions suggestive of Piroplasmida infection in erythrocytes. Inclusions showed the classical forms of ‘Maltese cross’ (Figure 6), with the formation of tetrads. The length obtained for the tetrads was 1.42 μm.

### 3.8. Description of a New Species: Theileria terrestris *nov. sp.*

DESCRIPTION

Taxonomic review

*Theileria terrestris* n. sp.

Type-host: Lowland tapir *Tapirus terrestris* (Mammalia: Peryssodactila).

Type-locality: Pantanal and Cerrado Biomes, Mato Grosso do Sul State, Brazil. (−19.301668883667233, −55.76404347032559; −21.583271686146354, −53.88629288988308).

Type-material: Holotype. A stained thin blood smear from a lowland tapir (*T. terrestris*–ID: PO-P) from Pantanal biome, containing the holotype (Figure 6) was deposited in the Vector-Borne Bioagents Laboratory of FCAV/UNESP (Jaboticabal, São Paulo, Brazil) in addition to genomic DNA samples extracted from the blood of lowland tapirs. Sample was collected and blood smear was made on 24 August 2018.

Vector: Unknow. It is assumed to be a local species of hard (Ixodida) tick.

Representative sequences: GenBank accession numbers: OP023828-OP0232835 (18S rRNA); OP169682, OP169600 and OP169601 (*cox*-1); OP376711, OP169596-OP169599 (*hsp70*).

Etymology: The species is named after it was encountered in *Tapirus terrestris* hosts from Pantanal and Cerrado Biomes in Brazil.

ZooBank reference numbers: pub: CCF171BB-BE8C-4B98-ABCB-1BAB5E8CFAC7; act: 5674F76B-EA24-46E9-B6CE-ADE9A3819273.

Description: This *Theileria* organism shows the classical inclusion form inside erythrocytes from *T. terrestris*, with tetrads of merozoites forming a “Maltese-cross”. It’s location inside the erythrocyte can be described as more peripherical when compared to *T. equi* inclusions, with a length size found of 1.42 μm. On fast-stained blood smears, it shows a purple-blue coloration. The occurrence of inclusions of different forms or sizes is unknown.

## 4. Discussion

Analyses conducted herein suggested an evident genetic separation between *T. equi* and the lowland tapir-related *Theileria* sp. detected in the present study, based on the near full-length 18S rRNA gene of Piroplasmida. The phylogenetic tree constructed using Bayesian inference and a total alignment size of 1460 bp showed a separation supported by high post probabilities values (100) between Clade VIII (*Equus* group) and *T. terrestris* clade. A clear separation was also observed on distance analysis performed using Splitstree software. These findings point out the existence of a potentially new *Theileria* species/phylogenetic group based on the Piroplasmida topology proposal [33]. The neighbor-joining tree constructed by Jalovecka et al. (2019) [33] with an alignment size of 1638 bp also achieved high bootstrap values (100) on separation of clades belonging to *Equus* group (Clade VIII), *Theileria* sensu stricto (Clade IX), and *Babesia* sensu stricto (Clade X) groups, which were the phylogenetically closest clades in their analysis. In our analysis, *Equus* group and *T. terrestris* clades fit more closely related to *Cytauxzoon* group (Clade VI) and distant from *Babesia* sensu stricto group. These differences may be due to the different sort of inferences used (neighbor-joining x Bayesian) as well as the fact that we included newly reported genotypes, which may influence the achieved topology. Indeed, *Theileria* and *Cytauxzoon* share some biological features. The sporozoites of both these representatives of the Theileriidae family are capable of invading hosts’ leukocytes during the schizogony phase followed by the invasion of red blood cells [1].

*Theileria* sp. in free-ranging lowland tapirs was reported in post-mortem blood and spleen samples from road-killed animals from the Pantanal biome (Mato Grosso State) [34]. When fragments of approximately 740 bp were analyzed by Bayesian phylogenetic inference, these sequences (GenBank access no. MZ491096, MZ490586) clustered together in a clade with *Theileria* sp. previously reported in cats from Brazil (GenBank access no. KP410270-KP410273, KF970930) [35,36] and closely related to an *T. equi* clade [34]. Unfortunately, near-full length 18S rRNA from *Theileria* genotypes from cats are not available, precluding a better assessment on the phylogenetic positioning of these sequences within Piroplasmida clades. Indeed, 18S rRNA sequences of *Theileria* sp. obtained from tapirs with approximately 400 bp already fit in a clade separately from *T. equi* in Bayesian analysis [5], but extended sequences would be significant to highlight the phylogenetic relationship of *Theileria* sp. obtained from tapirs with those genotypes or species obtained from other wild animals from Brazil.

The equid-associated *T. equi* is considered endemic in Brazil [8] and a causative of red blood cell destruction [37]. *Theileria equi* DNA has also been reported in different vertebrate hosts, such as dogs [38], sheep [39] and zebras [40]. Considering its potential capacity to infect a different range of hosts species, sequences retrieved from non-equid hosts that present high percentages of similarity rates on BLASTn analysis may mislead to incorrect taxonomic prediction as *T. equi* when based solely on this similarity. Previously, occurrence of *T. equi* was reported in lowland tapirs from Pantanal [4] and Amazon [5] biomes. For both studies, percentages of identity computed by BLASTn analyses were reported as 98% when comparing partial 18S rRNA gene sequences (ranging from 392 to 475 bp) of *Theileria* spp. from lowland tapirs with homologue sequences from *T. equi*. Although this value could be considered high, taxonomic positioning based on 18S rRNA partial sequences shows lower accuracy when compared to those performed with (near) complete gene [41], and extended 18S rRNA sequences provide more resolutive phylogenetic positioning of Piroplasmida [17,42,43].

Regarding the other target genes evaluated herein, the topology obtained by the *cox*-1-based phylogenetic tree was very similar to those obtained with 18S rRNA, with both analyses agreeing when it comes to positioning the *T. terrestris*-clade closely to, albeit separated from, Clade VIII (post probability value of 94). The use of mitochondrial sequences, as the *cox*-1 gene fragments, is a useful tool to resolve Piroplasmida phylogenetic topologies [16]. Indeed, some studies performed the concatenated analysis with both 18S rRNA and *cox*-1 to achieve even more informative topologies [16,44]. Unfortunately, once we are working with clinical samples from wild animals, which were collected in-field, it is difficult to concatenate genes from a single sample in a trustworthy way, once the occurrence of genetic diversity of *Theileria* members is reported in animals from the same population [45,46,47].

The *hsp70* gene from Piroplasmida codifies the heat shock protein 70 proteins. When using fragments from this gene to phylogenetic assess the relationship between *T. terrestris*-clade and other Piroplasmida clades, we observed that tapirs’ clade is now closely related to Clade IX (*Theileria* sensu stricto) and Clade X (*Babesia* sensu stricto) with high post-probabilities values (100). The pattern of evolution of a certain gene is an influent aspect on phylogenetic topologies [48]. Differences in the structure of the *hsp70* gene may reflect in distance between taxa once it codifies important functional proteins [49]. Even though the 18S rRNA is considered an important conservative gene for taxonomic positioning of Piroplasmida [33], the use of different nuclear and mitochondrial genes may answer questions about the evolutionary relationship among species from this Order.

Maltese-cross inclusion forms comprise four merozoites in one erythrocyte and are usual during the developmental stages of some Piroplasmida species [50]. This form has been described commonly in *Theileria* species, such as the horse-related species *T. equi, T. haneyi* [51], as well as in *T. parva* and in *Theileria* sp. from African waterbucks (*Kobus defassa*) [52]. Besides that, this inclusion form has also been reported for *Babesia microti* [50,53]. In the present study, Maltese-cross inclusions were found in the blood smear of one infected tapir. The length of the inclusion found in the blood smear of a *Theileria*-positive tapir from the present study was smaller (1.42 μm) than mean values reported for *T. equi* (1.88 μm) and larger than mean values of *T. haneyi* (1.15 μm) [51].

## 5. Conclusions

Phylogenetic analyses based on near-full length sequences of the 18S rRNA, *hsp70*, and *cox-1* genes supported the description of *Theileria terrestris* nov. sp. in tapir blood samples, which was positioned apart from all other *Theileria* species. *Theileria terrestris* nov. sp. represents a new Piroplasmida clade, yet to be characterized regarding biological features, vectors involved in the transmission cycles, additional vertebrate hosts, and pathogenicity.

## Figures and Tables

**Table 1:** Conventional and nested PCR protocols used in the present study.

Target Gene	Primers	Fragment Size (bp)	References
18S rRNA	1st reaction: BTF1 (5′-GGCTCATTACAACAGTTATAG-3′) and BTR21 (5′-CCCAAAGACTTTGATTTCTCTC-3′); 2nd reation: BTF2 (5′-CCGTGCTAATTGTAGGGCTAATAC-3′) and BTR2 (5′-GGACTACGACGGTATCTGATCG-3′).	~800	[13]
18S rRNA	Nbab_1F (5′- AAGCCATGCATGTCTAAGTATAAGCTTTT-3′) and 18Sapir (5′-GGATCACTCGATCGGTAGGAG-3′)	~1500	[23,24]
18S rRNA	1st reaction: Piro0F (5′-GCCAGTAGTCATATGCTTGTGTTA-3′) and Piro6R (5′-CTCCTTCCTYTAAGTGATAAGGTTCAC-3′); 2nd reaction: Piro1F (5′-CCATGCATGTCTWAGTAYAARCTTTTA-3′) and Piro5.5R (5′-CCTYTAAGTGATAAGGTTCACAAAACTT-3′)	~1500	[22]
*cox*-1	1st reaction: Bab_for1 (5′-ATWGGATTYTATATGAGTAT-3′) and Bab_Rev1 (5′-ATAATCWGGWATYCTCCTTGG-3′), Bab_for2; 2nd reation: (5′-TCTCTWCATGGWTTAATTATGATAT-3′) and Bab_Rev2 (5′-TAGCTCCAATTGAHARWACAAAGTG-3′)	~924	[14,15]
*cox-3*	COX3F (5′-ACTGTCAGCTAAAACGTATC-3′) and COX3R (5′-ACAGGATTAGATACCCTGG-3′)	~600	[16,17]
*hsp70*	hsp70F1 (5′-CATGAAGCACTGGCCHTTCAA-3′) and hsp70R2 (5′-GBAGGTTGTTGTCCTTVGTCAT-3)	~740	[18]
*cytb*	cytbF (5′-TTAGTGAAGGAACTTGACAGGT-3′) and cytbR (5′-CGGTTAATCTTTCCTATTCCTTACG-3′)	~1000	[16,17]
*β-tubulin*	Tubu-63F (5′-CAAATWGGYGCMAARTTYTGGGA-3′) and Tubu-3F (5′-TCGTCCATACCTTCWCCSGTRTACCAGTG-3′)	~1200	[19]
ITS-1	1st reaction: ITS15C (5′-CGATCGAGTGATCCGGTGAATTA-3′) and ITS13B (5′-GCTGCGTCCTTCATCGTTGTG-3′); 2nd reaction: (5′-AAGGAAGGAGAAGTCGTAACAAGG-3′) and ITS15C (5′-TTGTGTGAGCCAAGACATCCA-3′)	~450	[20]
*ema*-1	1st reaction: EMAE-F (5′-CCGCCCTTCACCTCGTTCTCAA-3′) and EMAE-R (5′-TCTCGGCGGCATCCTTGACCTC-3′); 2nd reaction: EMAI-F (5′-CCGTCTCCGTTGACTTGGCCG-3′) and EMAIR (5′-GGACGCGCTTGCCTGGAGCCT-3′)	~396	[21]

**Table 2:** Set of primers used for the sequencing of near-full length 18S rRNA gene.

Primers	Primers	Annealing Temperature	Primers Reference
Pair 1	Piro1F (5′-CCA TGC ATG TCT WAG TAY AAR CTT TTA-3′) and Piro5.5R (5′-CCT YTA AGT GAT AAG GTT CAC AAA ACT T-3′)	59 °C	[26]
Pair 2	BabF2 (5′-CCG TGC TAA TTG TAG GGC TAA TAC A-3′) and BabR2 (5′-GCT TGA AAC ACT CTA RTT TTC TCA A-3′)	59 °C	[18]
Single 1	Bab2F2 (5′-CTT TGA GAA ATT AGA GTG TTT-3′)	59 °C	Present study

## Data Availability

GenBank (http://www.ncbi.nlm.nih.gov/genbank) accession numbers: OP023828-OP0232835 (18S rRNA); OP169682, OP169600 and OP169601 (*cox*-1); OP376711, OP169596-OP169599 (*hsp70*).

## Supplementary Materials

The following supporting information can be downloaded at: https://www.mdpi.com/article/10.3390/microorganisms10122319/s1, File S1: Detailed reagent concentrations and thermal conditions of all protocols used in the present study.; File S2: Positive samples for detection of partial 18S rRNA from Piroplasmida: sample identification, biome where the animal was sampled, sampling date, gender and age.; File S3: List of tapirs that were sampled more than once and that presented at least one positive sample for Piroplasmida partial 18S rRNA amplification.; File S4: BLASTn analysis of the obtained gene fragments of Piroplasmida detected in lowland tapirs’ blood samples.
