# Supplementary material for: Theileria terrestris nov. sp.: A Novel Theileria in Lowland Tapirs (Tapirus terrestris) from Two Different Biomes in Brazil
Source: Microorganisms. 2022 Nov 23;10(12):2319. doi: 10.3390/microorganisms10122319 (PMC9784709; doi:10.3390/microorganisms10122319)
Supplement: Supplementary file 1 [file microorganisms-10-02319-s001.zip › microorganisms-1964825-supplementary.pdf]

Supplementary File S1. Detailed reagent concentrations and thermal conditions of all protocols used in the present study.

Target gene	Primers	Fragment size (bp)	Reagents concentrations	Thermal conditions	References
18S rRNA	1st reaction: BTF1 (5′-GGCTCATTACAACAGTTATAG-3′) and BTR21 (5′-CCCAAAGACTTTGATTTCTCTC-3′); 2nd reation: BTF2 (5′-CCGTGCTAATTGTAGGGCTAATAC-3′) and BTR2 (5′- GGACTACGACGGTATCTGATCG-3′).	~800	1st reaction: 10X Buffer, 50 mM $MgCl_2$, 10 mM dNTP, primers at 10 mM, 5U/ μL Platinum Taq DNA Polymerase (Life Technologies®, Carlsbad, California, USA), ultra-purified water and 5 μL from template DNA. 2nd reaction: same concentrations and 1 uL from amplified product from 1st reaction as template.	1st round: Initial denaturation at 93° C for 03 min, followed by 45 cycles of denaturation at 95° C for 30 sec, annealing at 58° C for 20 sec and extension at 72° C for 30 sec, and final extension at 72° C for 07 min. 2nd round: same conditions but anneling at 62° C for 20 sec.	[13]
18S rRNA	Nbab_1F (5′-AAGCCATGCATGTCTAAGTATAAGCTTTT-3′) and 18Sapir (5′-GGATCACTCGATCGGTAGGAG-3′)	~1,500	10X Buffer, 50 mM $MgCl_2$, 10 mM dNTP, primers at 10 mM, 5U/ μL Platinum Taq DNA Polymerase (Life Technologies®, Carlsbad, California, USA), ultra-purified water and 5 μL from template DNA.	Initial denaturation at 95° C for 03 min, followed by 35 cycles of denaturation at 94° C for 30 sec, annealing at 60° C for 01 min and extension at 72° C for 01 min, and final extension at 72° C for 04 min.	[23,24]
18S rRNA	1st reaction: Piro0F (5′-GCCAGTAGTCATATGCTTGTGTTA-3′) and Piro6R (5′-CTCCTTCCTYTAAGTGATAAGGTTCAC-3′); 2nd reaction: Piro1F (5′-CCATGCATGTCTWAGTAYAARCTTTTA-3′) and Piro5.5R (5′-CCTYTAAGTGATAAGGTTCACAAAACTT-3′)	~1,500	1st reaction: 10X Buffer, 50 mM $MgCl_2$, 10 mM dNTP, primers at 10 mM, 5U/ μL Platinum Taq DNA Polymerase (Life Technologies®, Carlsbad, California, USA), ultra-purified water and 5 μL from template DNA. 2nd reaction: same concentrations and 1	For both rounds: Initial denaturation at 95° C for 03 min, followed by 33 cycles of denaturation at 95° C for 01 min, annealing at 59° C for 01 min and extension at 72° C for 02 min, and	[22]

			uL from amplified product from 1st reaction as template.	final extension at 72º C for 10 min.	
cox-1	1st reaction: Bab_for1 (5′-ATWGGATTYTATATGAGTAT-3′) and Bab_Rev1 (5′- ATAATCWGGWATYCTCCTTGG- 3′), Bab_for2; 2nd reation: (5′-TCTCTWCATGGWTTAATTATGATAT-3′) and Bab_Rev2 (5′-TAGCTCCAATTGAHARWACAAAGTG-3′)	~924	1st reaction: 10X Buffer, 50 mM $MgCl_2$, 10 mM dNTP, primers at 10 mM, 5U/ µL Platinum Taq DNA Polymerase (Life Technologies®, Carlsbad, California, USA), ultra-purified water and 5 µL from template DNA. 2nd reaction: same concentrations and 1 uL from amplified product from 1st reaction as template.	1st round: Initial denaturation at 95º C for 01 min, followed by 35 cycles of denaturation at 95º C for 15 sec, annealing at 48,1º C for 30 sec and extension at 72º C for 30 sec, and final extension at 72º C for 01 min. 2nd round: same conditions but anneling at 52º C for 30 sec.	[14,15]
cox-3	COX3F (5′-ACTGTCAGCTAAAACGTATC-3′) and COX3R (5′-ACAGGATTAGATACCCTGG-3′)	~600	10X Buffer, 50 mM $MgCl_2$, 10 mM dNTP, primers at 10 mM, 5U/ µL Platinum Taq DNA Polymerase (Life Technologies®, Carlsbad, California, USA), ultra-purified water and 5 µL from template DNA.	Initial denaturation at 94º C for 05 min, followed by 40 cycles of denaturation at 94º C for 20 sec, annealing at 55º C for 30 sec and extension at 68º C for 01 min, and final extension at 72º C for 07 min.	[16,17]
hsp70	hsp70F1 (5′- GAAGCACTGGCCHTTCAA-3′) and hsp70R2 (5′-GBAGGTTGTTGTCCTTVGTCAT-3)	~740	10X Buffer, 50 mM $MgCl_2$, 10 mM dNTP, primers at 10 mM, 5U/ µL Platinum Taq DNA Polymerase (Life Technologies®, Carlsbad, California, USA), ultra-purified water and 5 µL from template DNA.	Initial denaturation at 95º C for 05 min, followed by 35 cycles of denaturation at 95º C for 20 sec, annealing at 60º C for 30 sec and extension at 72º C for 30 sec, and final extension at 72º C	[18]

				for 07 min.	
cytb	cytbF (5′-TTAGTGAAGGAACTTGACAGGT-3′) and cytbR (5′-CGGTTAATCTTTCCTATTCCTTACG-3′)	~1.000	10X Buffer, 50 mM $MgCl_2$, 10 mM dNTP, primers at 10 mM, 5U/ μL Platinum Taq DNA Polymerase (Life Technologies®, Carlsbad, California, USA), ultra-purified water and 5 μL from template DNA.	Initial denaturation at 94° C for 05 min, followed by 49 cycles of denaturation at 95° C for 20 sec, annealing at 56° C for 30 sec and extension at 68° C for 45 sec, and final extension at 72° C for 07 min.	[16,17]
β-tubulin	Tubu-63F (5′-CAAATWGGYGCMAARTTYTGGGA-3′) and Tubu-3F (5′-TCGTCCATACCTTCWCCSGTRTACCAGTG-3′)	~1.200	10X Buffer, 50 mM $MgCl_2$, 10 mM dNTP, primers at 10 mM, 5U/ μL Platinum Taq DNA Polymerase (Life Technologies®, Carlsbad, California, USA), ultra-purified water and 5 μL from template DNA.	Initial denaturation at 95° C for 05 min, followed by 30 cycles of denaturation at 94° C for 40 sec, annealing at 55° C for 1 min and extension at 72° C for 45 sec, and final extension at 72° C for 05 min.	[19]
ITS-1	1st reaction: ITS15C (5′-CGATCGAGTGATCCGGTGAATTA-3′) and ITS13B (5′-GCTGCGTCCTTCATCGTTGTG-3′); 2nd reaction: (5′-AAGGAAGGAGAAGTCGTAACAAGG-3′) and ITS15C (5′-TTGTGTGAGCCAAGACATCCA-3′)	~450	1st reaction: 10X Buffer, 50 mM $MgCl_2$, 10 mM dNTP, primers at 10 mM, 5U/ μL Platinum Taq DNA Polymerase (Life Technologies®, Carlsbad, California, USA), ultra-purified water and 5 μL from template DNA. 2nd reaction: same concentrations and 1 uL from amplified product from 1st reaction as template.	1st round: Initial denaturation at 94° C for 01 min, followed by 35 cycles of denaturation at 94° C for 30 sec, annealing at 52° C for 01 min and extension at 72° C for 01 min, and final extension at 72° C for 05 min. 2nd round: same	[20]

				conditions but anneling at 49° C for 1 min.	
ema-1	1st reaction: EMAE-F (5′-CCGCCCTTCACCTCGTTCTCAA-3′) and EMAE-R (5′-TCTCGGCGGCATCCTTGACCTC-3′); 2nd reaction: EMAI-F (5′-CCGTCTCCGTTGACTTGGCCG-3′) and EMAIR (5′- GGACGCGCTTGCCTGGAGCCT-3′)	~396	1st reaction: 10X Buffer, 50 mM $MgCl_2$, 10 mM dNTP, primers at 10 mM, 5U/ μL Platinum Taq DNA Polymerase (Life Technologies®, Carlsbad, California, USA), ultra-purified water and 5 μL from template DNA. 2nd reaction: same concentrations and 1 uL from amplified product from 1st reaction as template.	For both rounds: 1st round: Initial denaturation at 94° C for 04 min, followed by 39 cycles of denaturation at 94° C for 01 min, annealing at 60° C for 01 min and extension at 72° C for 01 min, and final extension at 72° C for 04 min.	[21]

Supplementary File S2. Positive samples for detection of partial 18S rRNA from Piroplasmida: sample identification, biome where the animal was sampled, sampling date, gender and age.

ID	Biome	Sampling date	Gender	Age
SO-C-1	Cerrado	11 April 2018	Male	Adult
RER-C	Cerrado	15 March 2016	Male	Sub-adult
NAI-C	Cerrado	03 May 2017	Female	Sub-adult
ANO-C-1	Cerrado	19 October 2016	Male	Adult
JHA-C	Cerrado	08 April 2016	Female	Sub-adult
CBI-C-1	Cerrado	23 February 2017	Female	Adult
ISA-C	Cerrado	16 March 2016	Female	Adult
AN-C	Cerrado	25 May 2017	Male	Sub-adult
CRO-C	Cerrado	25 June 2017	Male	Sub-adult
TI-C	Cerrado	12 March 2016	Male	Adult
PO-P	Pantanal	24 August 2018	Male	Sub-adult
SKI-P	Pantanal	14 June 2018	Male	Adult
MA-P-1	Pantanal	28 July 2013	Female	Adult
JO-P-1	Pantanal	27 October 2014	Male	Sub-adult
CO-P	Pantanal	23 July 2013	Male	Sub-adult
CRA-P-1	Pantanal	21 July 2013	Female	Adult
JA-P	Pantanal	05 May 2014	Female	Sub-adult
XS-P	Pantanal	21 August 2017	Male	Sub-adult
IA-P	Pantanal	10 December 2017	Male	Adult
COS-P	Pantanal	08 December 2017	Male	Sub-adult
SIA-P	Pantanal	05 December 2017	Female	Sub-adult
DA-P	Pantanal	25 August 2018	Female	Sub-adult

NAO-P	Pantanal	20 June 2016	Male	Adult
RTA-P	Pantanal	10 May 2015	Female	Adult
RCK-P	Pantanal	21 October 2014	Male	Adult
WEP-P-1	Pantanal	14 November 2015	Female	Sub-adult
HRY-P	Pantanal	22 August 2016	Male	Sub-adult
VA-P-1	Pantanal	01 September 2018	Female	Sub-adult
FPE-P	Pantanal	17 December 2014	Male	Adult
RO-P	Pantanal	25 June 2018	Male	Sub-adult
LRO-P	Pantanal	10 December 2017	Male	Adult
SCO-P	Pantanal	08 December 2016	Male	Adult
MLE-P	Pantanal	03 July 2014	Female	Sub-adult
BEN-P	Pantanal	16 August 2016	Male	Adult
GIL-P	Pantanal	27 October 2017	Male	Sub-adult
FFO-P-1	Pantanal	29 August 2018	Male	Adult
MIA-P	Pantanal	25 June 2018	Female	Sub-adult
DHO-P-1	Pantanal	26 October 2017	Male	Sub-adult
CHA-P-2	Pantanal	22 October 2018	Female	Sub-adult
NLA-P	Pantanal	25 June 2018	Female	Adult
KIN-P	Pantanal	12 July 2014	Female	Adult
PNA-P	Pantanal	15 November 2015	Female	Adult
GAO-P	Pantanal	19 June 2018	Male	Adult
INA-P	Pantanal	27 August 2018	Female	Adult
MCI-P	Pantanal	02 July 2015	Female	Adult
GLA-P	Pantanal	06 July 2014	Female	Sub-adult
CNA-C-2	Cerrado	19 September 2018	Female	Adult
SO-C-2	Cerrado	09 February 2017	Male	Adult
FFO-P-2	Pantanal	23 August 2017	Male	Adult
LNA-P-2	Pantanal	11 August 2018	Female	Adult
VA-P-2	Pantanal	19 August 2017	Female	Sub-adult
DHO-P-2	Pantanal	25 June 2018	Male	Sub-adult
JE-P-2	Pantanal	09 June 2016	Male	Sub-adult
BRS-P	Pantanal	11 June 2016	Male	Sub-adult
TD-P-2	Pantanal	17 December 2014	Male	Sub-adult
TD-P-3	Pantanal	18 June 2016	Male	Sub-adult
WE-P-2	Pantanal	18 June 2016	Female	Sub-adult
WE-P-3	Pantanal	15 December 2016	Female	Sub-adult
SAO-P-2	Pantanal	20 October 2013	Male	Adult
SAO-P-3	Pantanal	11 May 2015	Male	Adult
NEC09-C	Cerrado	04 April 2016	Female	Sub-adult
NEC07-C	Cerrado	10 March 2016	Female	Sub-adult
NEC15-C	Cerrado	20 July 2016	Male	Adult
NEC24-C	Cerrado	18 November 2016	Female	Adult

Supplementary File S3. List of tapirs that were sampled more than once and that presented at least one positive sample for Piroplasmida partial 18S rRNA amplification.

Animal ID	Biome	Sample ID	Sampling dates	Partial 18S rRNA PCR
SO-C	Cerrado	SO-C-1	11 April 2018	Positive
		SO-C-2	09 February 2017	Positive
ANO-C	Cerrado	ANO-C-1	19 October 2016	Positive
		ANO-C-2	28 July 2017	Negative
CBI-C	Cerrado	CBI-C-1	23 February 2017	Positive
		CBI-C-2	11 October 2018	Negative
CNA-C	Cerrado	CNA-C-1	05 April 2016	Negative
		CNA-C-2	19 September 2018	Positive
CHA-P	Pantanal	CHA-P-1	22 August 2017	Negative
		CHA-P-2	22 October 2018	Positive
SAO-P	Pantanal	SAO-P-1	29 October 2017	Negative
		SAO-P-2	20 October 2013	Positive
		SAO-P-3	11 May 2015	Positive
MA-P	Pantanal	MA-P-1	28 July 2013	Positive
		MA-P-2	19 May 2015	Negative
JE-P	Pantanal	JE-P-1	06 December 2017	Negative
		JE-P-2	09 June 2016	Positive
TD-P	Pantanal	TD-P-1	16 November 2015	Negative
		TD-P-2	17 December 2014	Positive
		TD-P-3	18 June 2016	Positive
JO-P	Pantanal	JO-P-1	27 October 2014	Positive
		JO-P-2	20 August 2016	Negative
CRA-P	Pantanal	CRA-P-1	21 July 2013	Positive

		CRA-P-2	18 October 2014	Negative
LNA-P	Pantanal	LNA-P-1	29 August 2017	Negative
		LNA-P-2	11 August 2016	Positive
WE-P	Pantanal	WE-P-1	14 November 2015	Positive
		WE-P-2	16 June 2016	Positive
		WE-P-3	15 December 2016	Positive
VA-P	Pantanal	VA-P-1	01 September 2018	Positive
		VA-P-2	19 August 2017	Positive
FFO-P	Pantanal	FFO-P-1	29 August 2018	Positive
		FFO-P-2	23 August 2017	Positive
PNA-P	Pantanal	PNA-P-1	15 November 2015	Positive
		PNA-P-2	25 August 2017	Negative
DHO-P	Pantanal	DHO-P-1	26 October 2017	Positive
		DHO-P-2	25 June 2018	Positive

Supplementary File S4. BLASTn analysis of the obtained gene fragments of Piroplasmida detected in lowland tapirs' blood samples.

**Gene Fragment (size)**	**Sample ID**	**GenBank accession number**	**Biome**	**BLASTn Best Match**	***Query cover***	***E-value***	**Similarity rate**
hsp70 (782 bp)	JHA-C	OP376711	Cerrado	*Theileria equi* – Mongolia (AB248743)	99%	0.0	81.69%
hsp70 (771bp)	JA-P	OP169598	Pantanal	*Theileria equi* – Mongolia (AB248743)	100%	0.0	81.17%

hsp70 (687 bp)	DA-P	OP169599	Pantanal	*Theileria equi* – Mongolia (AB248743)	99%	0.0	81.49%
hsp70 (695 bp)	RO-P	OP169597	Pantanal	*Theileria equi* – Mongolia (AB248743)	99%	0.0	81.36%
hsp70 (694 bp)	FFO-P-1	OP169596	Pantanal	*Theileria equi* – Mongolia (AB248743)	99%	0.0	81.07%
cox1 (410 bp)	JHA-C	OP169682	Cerrado	*Babesia bigemina* – China (JQ518300)	88%	$1,00^{-46}$	77.11%
cox1 (354 bp)	DA-P	OP9601	Pantanal	*Babesia bigemina* – China (JQ518300)	95%	$6,00^{-39}$	76.45%
cox1 (357 bp)	FFO-P-1	OP9600	Pantanal	*Babesia bigemina* – China (JQ518300)	94%	$1,00^{-36}$	76.09%
18S rRNA (1,401 bp)	JHA-C	OP023833	Cerrado	*Theileria* sp. – Kenia (KF597074)	100%	0.0	95.23%
18S rRNA (1,518 bp)	JA-P	OP023832	Pantanal	*Theileria equi* – Brazil (MG052902)	100%	0.0	95.33%
18S rRNA	XS-P	OP023830	Pantanal	*Theileria equi* - Israel	100%	0.0	95.48%

(1,348 bp)				(MN611344)			
18S rRNA (1,213 bp)	DA-P	OP023831	Pantanal	*Theileria equi* - Chile (MT463613)	100%	0.0	95.31%
18S rRNA (1,182 bp)	RO-P	OP023828	Pantanal	*Theileria equi* - Israel (MN611344)	100%	0.0	95.53%
18S rRNA (1,308 bp)	FFO-P-1	OP023829	Pantanal	*Theileria equi* – USA (CP001669)	100%	0.0	95.40%
18S rRNA (1,473 bp)	PO-P	OP023834	Pantanal	*Theileria equi* – USA (CP001669)	100%	0.0	95.40%
18S rRNA (1,352 bp)	JO-P-1	OP023835	Pantanal	*Theileria equi* – Israel (MN611343)	100%	0.0	95.44%
